# Costing and Cost-Effectiveness of a Mobile Health Intervention (ImTeCHO) in Improving Infant Mortality in Tribal Areas of Gujarat, India: Cluster Randomized Controlled Trial

**DOI:** 10.2196/17066

**Published:** 2020-10-14

**Authors:** Dhiren Modi, Somen Saha, Prakash Vaghela, Kapilkumar Dave, Ankit Anand, Shrey Desai, Pankaj Shah

**Affiliations:** 1 Society for Education, Welfare and Action (SEWA) Rural Bharuch India; 2 Indian Institute of Public Health Gandhinagar Gandhinagar India; 3 Health & Family Welfare Department, Gujarat Gandhinagar India

**Keywords:** mHealth, cost-effectiveness, life-years saved, India, ASHA

## Abstract

**Background:**

During 2013, a mobile health (mHealth) program, Innovative Mobile Technology for Community Health Operation (ImTeCHO), was launched in predominantly tribal and rural communities of Gujarat, India. ImTeCHO was developed as a job aid for Accredited Social Health Activists (ASHAs) and staff of primary health centers to increase coverage of maternal, neonatal, and child health care.

**Objective:**

In this study, we assessed the incremental cost per life-years saved as a result of the ImTeCHO intervention as compared to routine maternal, neonatal, and child health care programs.

**Methods:**

A two-arm, parallel, stratified cluster randomized trial with 11 clusters (primary health centers) randomly allocated to the intervention (280 ASHAs, n=2,34,134) and control (281 ASHAs, n=2,42,809) arms was initiated in 2015 in a predominantly tribal and rural community of Gujarat. A system of surveillance assessed all live births and infant deaths in the intervention and control areas. All costs, including those required during the start-up and implementation phases, were estimated from a program perspective. Incremental cost-effectiveness ratios were estimated by dividing the incremental cost of the intervention with the number of deaths averted to estimate the cost per infant death averted. This was further analyzed to estimate the cost per life-years saved for the purpose of comparability. Sensitivity analysis was undertaken to account for parameter uncertainties.

**Results:**

Out of a total of 5754 live births (3014 in the intervention arm, 2740 in the control arm) reported in the study area, per protocol analysis showed that the implementation of ImTeCHO resulted in saving 11 infant deaths per 1000 live births in the study area at an annual incremental cost of US $163,841, which is equivalent to US $54,360 per 1000 live births. Overall, ImTeCHO is a cost-effective intervention from a program perspective at an incremental cost of US $74 per life-years saved or US $5057 per death averted. In a realistic environment with district scale-up, the program is expected to become even more cost-effective.

**Conclusions:**

Overall, the findings of our study strongly suggest that the mHealth intervention as part of the ImTeCHO program is cost-effective and should be considered for replication elsewhere in India.

**Trial Registration:**

Clinical Trials Registry of India CTRI/2015/06/005847; http://www.ctri.nic.in/Clinicaltrials/pdf_generate.php?trialid=11820&EncHid=&modid=&compid=%27,%2711820det%27

## Introduction

The National Health Mission of 2005 introduced a cadre of village-based frontline health worker communities named Accredited Social Health Activists (ASHAs) to facilitate the delivery of proven community-based maternal, neonatal, and child health (MNCH) services in rural areas of India, with one ASHA for every 1000 individuals. Despite this initiative, the coverage of selected MNCH services aimed at reducing mortality and undernutrition has remained low [1,2]. Coverage of key MNCH services and outcomes are inequitably distributed among the states of India [3]. The tribal communities have worse health indicators compared to those of nontribal communities [4]. The reasons for this low coverage are inadequate institutional capacity for optimal supervision and support to ASHAs in addition to insufficient skills, poor quality of training, and complexity of tasks to be performed [5,6].

Mobile telecommunication technology for health or mobile health (mHealth) has emerged as an important tool for global health. Its potential for improving the coverage and outcome of MNCH services through enhancing performance of frontline health workers has been well documented [7,8]. Despite its promise and potential, the Global Observatory for eHealth survey found that most initiatives on mHealth have not expanded beyond small-scale pilot projects [9]. In addition, a World Bank report on mobile apps for the health sector found that evidence of mHealth was limited, particularly for moving beyond intermediate outcomes to better health, especially in rural settings [10]. There is a gap in terms of rigor, intervention type, measurement of effectiveness, and, more importantly, cost-effectiveness of mHealth programs as well as detailed discussion on the scale-up of mHealth interventions.

An mHealth-based intervention named Innovative Mobile Technology for Community Health Operation (ImTeCHO) was designed for predominantly tribal and rural communities of Gujarat, India. ImTeCHO was developed as a job aid for ASHAs and staff of primary health centers to increase the coverage of MNCH care. A cluster randomized controlled trial was carried out to assess the effectiveness of the ImTeCHO intervention between 2015 to 2018, details of which have been published elsewhere [11]. In the present study, we assessed the incremental cost per life-year saved as a result of the ImTeCHO intervention as compared to routine MNCH programs.

## Methods

### Study Design

In 2015, a voluntary organization named Society for Education, Welfare and Action-Rural (SEWA-Rural) in active partnership with the government of Gujarat and the software partner Argusoft India Ltd began evaluating the effectiveness of the ImTeCHO intervention over a 3-year period through a cluster randomized controlled trial to improve the delivery of proven MHCH services through community-based ASHAs by enhancing their motivation and strengthening supervision in tribal areas of Gujarat, India. This trial was conducted within the existing public health system involving 22 primary health centers serving a total population of 4,76,943 individuals. Eleven primary health centers (280 ASHAs, n=2,34,134) were randomized to the intervention arm and 11 were randomized to the control arm (281 ASHAs, n=2,42,809). The control arm continued to receive usual health services from the government and other providers, while the intervention arm received the ImTeCHO intervention. There were four components of the ImTeCHO intervention:

1. Scheduling and task management: ASHAs received reminders on their mobile phone apps regarding the services to be provided every day.
2. Health promotion using multimedia: nine mobile-based short videos assisted ASHAs to provide counseling about key healthy behaviors during their home visits to households.
3. Decision support system screening, risk stratification, and treatment: the ImTeCHO app showed a diagnosis and customized treatment plan based on entry provided by the ASHAs on a mobile phone.
4. Support and supervision: the ImTeCHO web interface provided tools and real-time information to medical officers for offering timely support and supervision. ImTeCHO integrated a checklist (to ensure standardization of services) with other features offered through mobile technology, such as the ability to transfer data instantly and apply an algorithm automatically to data entered, along with features to ensure check-and-balance for truthfulness and accuracy of the collected information.

ASHAs from the control and intervention areas received a one-time refresher training on Home-based Newborn Care and Integrated Management of Neonatal and Childhood Illnesses. This was necessitated for ethical reasons as well as to ensure that ASHAs from both the intervention and control arms were updated about newer guidelines on the management of childhood illness to attribute effectiveness to the ImTeCHO intervention itself instead of only training.

Along with the intervention effectiveness, efforts were made to measure the incremental cost-effectiveness ratio of the ImTeCHO intervention. Estimates of intervention cost-effectiveness were calculated for different scenarios.

### Framework for Assessing the Incremental Cost of Delivering the ImTeCHO Intervention

Cost data were collected from a program perspective. As the intervention was delivered at the doorstep and part of routine home visits of ASHAs, there were insignificant, if any, costs incurred by the households. Hence, costing from a program perspective was deemed appropriate. An incremental costing approach was adopted for the present study. The incremental costing approach takes into consideration the difference in cost and the additional benefits incurred on implementing the intervention compared with the routine case scenario (control arm). An incremental costing approach involves collecting additional financial costs, representing actual monetary flow on the goods and services purchased for delivering the program from a provider perspective. This type of costing enabled an analysis of cost by different program phases and program activities, and the unit cost of the program. Following the World Health Organization guide to cost-effectiveness analysis, all research costs such as field testing and data collection were not included in the calculation of costs [12].

Two cost heads, start-up cost and annual implementation cost, were included in the 2016-2017 price. Cost data collection included a study of financial records of the ImTeCHO project. Start-up costs included those associated with activities conducted during the preimplementation period prior to February 2016, such as expenses for training, and a one-time capital cost that included software development. Capital costs, including start-up costs such as software development, were estimated by completing a checklist of all equipment (such as mobile phones) and furniture used in the program, and the useful life of the equipment. The orientation training cost was assumed to last for 3 years. Refresher training was assumed to be a recurrent activity. All capital costs, including start-up costs, were annualized assuming a useful life year. Initial software development costs (excluding annual costs on maintenance and upgrades) were assumed to last for 10 years (the cost incurred on development is expected to last until strategies for the program remain in place); four-wheelers were assumed to last for 10 years; two-wheelers were assumed to last for 7 years; and laptops, projectors, and printers were assumed to last for 5 years. Mobile handsets were assumed to last for 3 years on average. The cost of capital items was annualized across the project life, with discounting at an annual rate of 3%. The data sources included program financial reports, interviews with key officials, and surveillance data.

In addition to capital costs, other fixed costs included the costs of development of guidelines for implementation and their adherence, training costs for both staff and supervisors, communication material costs, supplies, and an additional incentive to ASHAs. The staff involved in the project were asked to estimate the amount of time spent on various activities at different times of the intervention.

The intervention implementation period was from February 2016 to January 2017. Software maintenance, internet data plan, additional incentives for ASHAs, and salaries for supervisory staff were important annual expenditures during the implementation period.

Start-up costs and annual implementation costs were summed to determine the total cost. All costs are presented in US dollars. Costs were converted to constant values and are reported as an annualized cost at the 2016-2017 price.

### Surveillance to Collect Program Effectiveness Outcome Indicators: Infant Mortality

Infant deaths was the main outcome of interest to reflect program effectiveness for this study. To obtain complete information of all infant deaths, all pregnancy registrations, their outcomes, and survival status of all live births up to 1 year of age in all study clusters throughout the study period were counted as part of ongoing, prospective, pregnancy, and mortality surveillance. This was carried out by a data collection team who conducted a field survey of the entire study area every 3 months. One data collector covered approximately 25,000 individuals in the population. During their field surveys, the data collectors visited all localities of the study villages and met with ASHAs to register pregnant women who were native of the study village. All live births to women native to the study village were included, irrespective of place of the birth.

Once pregnancy was registered, the data collectors prospectively tracked pregnancy outcome and survival status up to 1 year after delivery by visiting the household at regular intervals. The data collectors recorded all maternal and infant deaths as part of the surveillance. Data entry was done in a customized, Android-based mobile phone app. The verbal autopsy method was used to review the cause of maternal and infant deaths [13]. When a data collector recorded a maternal or infant death, a supervisor visited the household of the deceased infant or mother, validated the death, and performed a verbal autopsy to determine the cause of death. A qualified, experienced doctor assigned the cause of death for each infant death after reviewing the verbal autopsy report. Supervisors also validated the surveillance data regarding the accuracy of pregnant registrations and pregnancy outcomes by calling random respondents over the phone and making random field visits. Deaths that were averted were calculated as the differences in deaths reported in the intervention and control arms from the surveillance activity.

### Statistical Analysis to Estimate the Incremental Cost-Effectiveness Ratio of the Program

Cost-effectiveness ratios were estimated by dividing the incremental cost of the intervention with the number of infant deaths averted to estimate the cost per infant death averted. As per World Bank Data, a life expectancy of 68.35 years (2016 value) [14] was assumed to estimate the number of life years saved by averting an infant death, and this value as a denominator provided the cost per life years saved. According to the most commonly cited cost-effectiveness thresholds, an intervention is considered to be cost-effective if the incremental cost-effectiveness ratio (cost per life years saved) is less than the per capita gross domestic product (GDP) [15].

Both intention to treat (ITT) and per-protocol (PP) analyses were used in this study. ITT involved all live births, including women who were not native to the village but gave birth there. In other words, ITT included all live births that occurred in the study villages. However, there is a local custom in which some of the pregnant women leave their in-laws’ homes during the last trimester and stay at their maternal home up to 2-3 months after delivery. These women and infants were partially exposed to the treatment arm. Therefore, PP analysis was performed by excluding deliveries of such women and infants to assess the effectiveness of the intervention only among those who were fully exposed.

A sensitivity analysis was conducted using the upper and lower estimates for various variables in the model to determine the impact of changes on cost per life years saved. A decision tree model was constructed to combine information from a wide variety of sources, extrapolate costs and health effects beyond the time period of the ImTeCHO study, and evaluate multiple potential interventions packaged into the strategies. Cost estimates that are relevant to the government staff in one representative district were estimated to assess the cost of scale up of such a program by the government. A sensitivity analysis for different program costs and effect estimates was conducted. The study report adhered to the Consolidated Health Economic Evaluation Reporting Standards (CHEERS) requirement for reporting the economic evaluation of health interventions [16]. The study protocol was approved by the SEWA-Rural Institutional Ethics Committee on January 29, 2016.

## Results

### Costs

The costs incurred in both the intervention and control arm were annualized based on the life span of the equipment. More than three-quarters (76%) of the cost was directed toward annual recurrent implementation costs, comprising personnel, training, software development, annual maintenance, supportive supervision, and monitoring costs. The item-specific annualized costs and the differences in costs for the intervention and control arms are detailed in Table 1.

**Table 1:** Annual start-up and implementation costs (US dollars) of the ImTeCHO^a^ program in the implementation and control arms from a program perspective.

Cost Category			Annualized cost in intervention arm		Age (%)		Annualized cost in control arm		Cost difference
**Annual start-up costs**									
	Total	45,647		26		17,789		27,858	
	Software development cost	7951		4.4		—^b^		7951	
	Vehicles	1135		0.6		—		1135	
	Mobile handset	11,873		6.5		—		11,873	
	Other IT^c^ equipment	397		0.2		—		397	
	Training cost	24,291		13.4		17,789		6,502	
**Annual implementation costs**									
	Total	126,405		74		—		126,405	
	Personnel	24,919		14.5		—		24,919	
	Training cost	256		0.1		—		256	
	Software annual development and maintenance	49,599		28.8		—		49,599	
	Travel	2123		1.2		—		2123	
	ASHA^d^ incentive	35,166		20.4		—		35,166	
	IT expense	12,935		7.5		—		12,935	
	Office expenses	1406		0.8		—		1406	
Total costs		172,052		100		17,789		154,263	

^a^IMTeCHO: Innovative Mobile Technology for Community Health Operation.

^b^—:not relevant; no costs incurred in the control arm.

^c^IT: information technology.

^d^ASHA: Accredited Social Health Activists.

### Valuation of Study Outcome

Implementation of the ImTeCHO intervention with 561 ASHAs across 22 primary health centers of Gujarat resulted in 11 infant deaths per 1000 live births averted in the PP analysis (infant mortality rate of 56.4 per 1000 live births in the intervention area as compared to 67.2 per 1000 live births in the control area). This implies a reduction of 16% infant deaths per-protocol in the study area (Table 2). This resulted in an increase in 735 life years, with a life expectancy of 68.35 years. Although the protective effect of the ImTeCHO intervention was observed across other indicators such as early neonatal mortality rate, neonatal mortality rate, and stillbirths, to avoid double counting, these were not accounted for in the present analysis.

**Table 2:** Surveillance data on study outcome variables from February 2016 to January 2017.

Variable	Intention to Treat			Per Protocol		
	Control	Intervention	Control		Intervention	
Number of live births	4059	4171	2740		3014	
Hospital deliveries (%)	83.5	80.3	80.8		77.1	
Number of early neonatal deaths	106	113	81		83	
Number of neonatal deaths	138	142	102		104	
Number of stillbirths	107	90	79		72	
Number of infant deaths	236	233	184		170	

### Cost-Effectiveness

**Figure 1:**
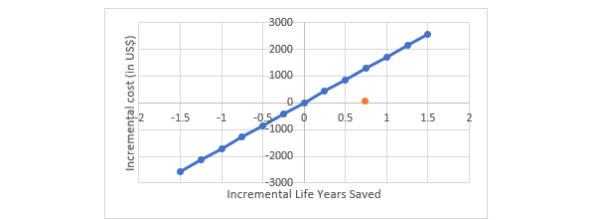
Cost-effectiveness plane with an incremental cost-effectiveness ratio, 2016-2017.

The implementation of ImTeCHO resulted in saving 11 infant deaths per 1000 live births in the study area at an annual incremental cost of US $54,360 per 1000 live births. Overall, ImTeCHO is a cost-effective intervention from a program perspective at an incremental cost of US $74 per life years saved or US $5057 per death averted (Table 3). At a per capita GDP of US $1709 in 2016, the ImTeCHO intervention is considered to be cost-effective (Figure 1).

**Table 3:** Cost-effectiveness of the ImTeCHO^a^ program.

Point estimate of infant mortality rate	Value
Total births in the study area (n)	3014
Cost per live birth (US $)	54
Cost per 1000 live births (US $)	54,360
Infant deaths averted per 1000 live births (n)	11
Life years saved (life expectancy: 68.35 years)	735
Cost per infant deaths averted (US $)	5057
Cost per life years saved due to infant deaths averted (US $)	74

^a^ImTeCHO: Innovative Mobile Technology for Community Health Operation.

### Sensitivity Analysis

To test the cost-effectiveness of the ImTeCHO program under different scenarios, we hypothesized three cases: (1) overall infant mortality rate reported in the study area, (2) estimated cost for district scale up with the actual ImTeCHO effect, and (3) estimated cost for district scale up with 50% of the reported ImTeCHO effect. The ImTeCHO program remained cost-effective across all scenarios (Table 4).

**Table 4:** Sensitivity analysis under different scenarios.

Variable			Value
**Reference Case (IMR^a^ per protocol in the study area)**			
	Infant deaths averted (n)	11	
	Cost per ASHA^b^ (US $)	579	
	Cost per infant death averted (US $)	5057	
	Cost per LYS^c^ (US $)	74	
**Scenario 1: IMR as intention-to-treat in the study area**			
	Infant deaths averted (n)	2	
	Cost per ASHA (US $)	578.95	
	Cost per infant death averted (US $)	17,225	
	Cost per LYS (US $)	252	
**Scenario 2: Wider district scale-up with 100% observed effectiveness**			
	Infant deaths averted (n)	11	
	Cost per ASHA (US $)	75	
	Cost per infant death averted (US $)	824	
	Cost per LYS (US $)	12	
**Scenario 3: Wider district scale-up with 50% observed effectiveness**			
	Infant deaths averted (n)	5	
	Cost per ASHA (US $)	75	
	Cost per infant death averted (US $)	1649	
	Cost per LYS (US $)	24	

^a^IMR: infant mortality rate.

^b^ASHA: Accredited Social Health Activists.

^c^LYS: life-years saved.

## Discussion

This economic evaluation study compared the costs and consequences of implementing an mHealth program (ImTeCHO) in the existing routine health services of a tribal block of Gujarat, India, compared to routine MNCH provided by frontline health workers (ASHAs). The findings are presented from a program perspective using an incremental cost approach. The per capita GDP of India at the 2016 price was US $1709 [17]. Overall, ImTeCHO is a cost-effective intervention from a program perspective at an incremental cost of US $74 per life years saved or US $5057 per death averted. Analysis under different scenarios showed that the program is expected to be even more cost-effective in a realistic environment with district scale up. The program is expected to be cost-effective even with a 50% reduction in effectiveness reported under the trial phase.

Our study used actual mortality data to report cost-effectiveness of an mHealth intervention. ReMiND, a nonrandomized study, implemented an mHealth app in 2012 through 259 ASHAs from two blocks of the Kaushambi district of the state of Uttar Pradesh in India, which resulted in a reduction of 0.2% maternal and 5.3% neonatal deaths. The incremental cost of the ReMiND program was US $205 per disability-adjusted life year averted or US $5865 per death averted [18]. However, the ReMiND study relied on modeling to estimate the number of deaths averted.

Implementation of an intervention as part of a routine government system has a distinct cost advantage for two reasons: first, the program could leverage the existing public infrastructure, and second, there is greater potential for the program to be replicated across the state of Gujarat, particularly in high-focused districts with a high infant mortality rate. The initial investment in software development, induction training, and supportive supervision were the key drivers for the success of the ImTeCHO program.

ImTeCHO, as a mobile phone app in the hands of health workers, has potential to bridge an important gap in the delivery of existing public health programs through ensuring data entry at the point of service delivery by the health provider through a handheld device, thereby ensuring better quality of data. This remains an area of future research once ImTeCHO is scaled up.

A limitation of our analysis is that we did not assess the health care input cost or time spent by health workers in training, supportive supervision by medical officers, and other supervisors from the health system. As a standard cost-effectiveness analysis, it was implicitly assumed that opportunity costs were equal and that it does not matter from which health care input the resources were drawn [19]. The ImTeCHO trial was conducted in partnership with the local health government, and thus, the health care input cost was borne by the state to aid in potential scale up of the intervention. As the program operated in a realistic environment, it was assumed that the health care input cost will be absorbed as part of the health budget for replication of the program. The total cost of implementing the intervention was assumed to be the same in PP and ITT analyses; however, the total cost incurred to deliver the intervention among less than 5 live births included in the PP analysis would have been lower. There is potential for the cost-effectiveness to decrease over time as other interventions designed to directly or indirectly enhance maternal health and lower infant mortality take effect. However, this remains an area of future research.

Overall, the findings of our study strongly suggest that the mHealth intervention as part of the ImTeCHO program is cost-effective and should be considered for replication elsewhere in India and beyond.

## Abbreviations

ASHA: Accredited Social Health Activists
GDP: gross domestic product
ImTeCHO: Innovative Mobile Technology for Community Health Operation
ITT: intention to treat
mHealth: mobile health
MNCH: maternal, neonatal, and child health care
PP: per protocol
SEWA-Rural: Society for Education, Welfare and Action-Rural
